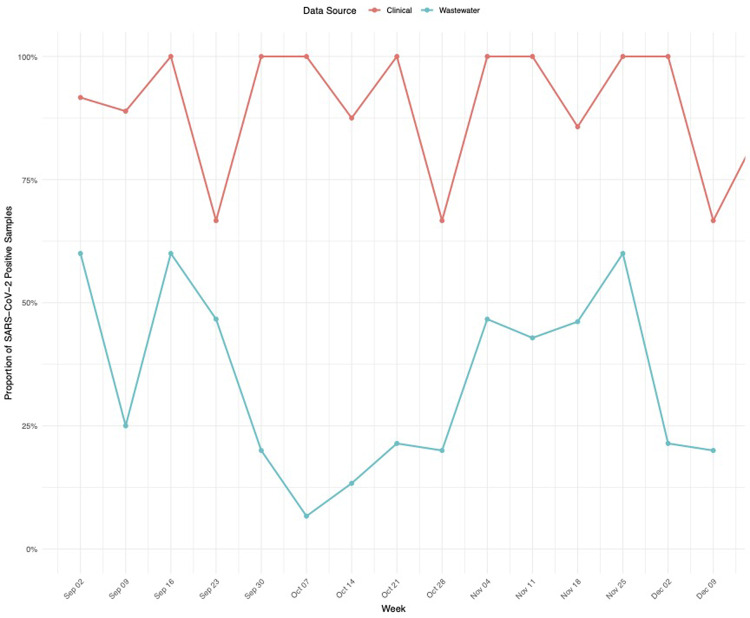# 239 COpAT Impact: Improving Care for Patients with Bone and Joint Infections with the Complex Outpatient Antimicrobial Therapy Program

**DOI:** 10.1017/ash.2026.10730

**Published:** 2026-06-23

**Authors:** Keyner Rojas, Nicole Stephenson, Windy Tanner, Amanda Darling, Hannah Healy, Inci Yildirim, Scott Roberts, Richard Martinello

**Affiliations:** 1 Yale University School of Public Health; 2 Ginkgo Bioworks; 3 Yale University

## Abstract

**Purpose:** Wastewater surveillance effectively monitors pathogens. This pilot study evaluated the feasibility and utility of hospital-level wastewater surveillance by integrating wastewater and electronic health record (EHR) data. Analyses focused on hospital-acquired infections (HAI) and temporal lags between wastewater and clinical detection. **Methods:** From August to December 2024, wastewater autosamplers operated across five hospital pavilions at Yale New Haven Hospital, collecting samples every five minutes during a 24-hour period three times per week. Samples were analyzed by dPCR for SARS-CoV-2, Influenza (A/B), and additional pathogens. Deidentified EHR data included admissions, diagnoses, and laboratory data. The primary focus was lab-confirmed HAI SARS-CoV-2 and Influenza. HAI was defined as infections diagnosed during hospitalization without evidence at admission. Clinical and wastewater data were used to calculate the proportion of positive samples, and correlation was assessed using Spearman’s rank correlation coefficient (rho). Correlations were evaluated for lagged associations across 1–3-week lags. A sensitivity analysis was conducted by including all SARS-CoV-2 encounters. **Results:** Among 33,579 patient encounters, 62 SARS-CoV-2 and 74 Influenza HAI encounters were identified. This corresponded to 97 and 148 tests for SARS-CoV-2 and Influenza, respectively. Of 187 wastewater samples collected 60 (31.1%) and 1 (0.5%) were positive for SARS-CoV-2 and Influenza respectively. Due to only a single detection of Influenza in the wastewater, correlation analysis was limited to SARS-CoV-2. A correlation test of the data found no statistically significant correlation between wastewater and clinical data when aligned temporally (rho: -0.21, p: 0.51). Lagged correlations between wastewater and clinical SARS-CoV-2 positivity were evaluated across 1–3-week temporal lags. These lagged correlations were not statistically significant, but rho increased in magnitude from a 1-week lag (rho: -0.04, p: 0.89) to a 3-weeks lag (rho: 0.46, p:0.21). The sensitivity analysis found no statistically significant correlation between wastewater and clinical positivity. **Conclusions:** Hospital-level wastewater surveillance shows potential as an early indicator of HAI SARS-CoV-2 infections, with exploratory trends suggesting a ~3-week lead time results in stronger associations between clinical and wastewater data. Although limited by small HAI sample sizes and a short wastewater sampling period, these findings support further evaluation in larger cohorts and highlights pathogen-specific limitations as observed for Influenza. Follow-up studies should employ longer wastewater sampling windows and further refine methods to account for community-associated SARS-CoV-2 contributions to hospital wastewater, an area of active investigation by our group.

**Figure:**
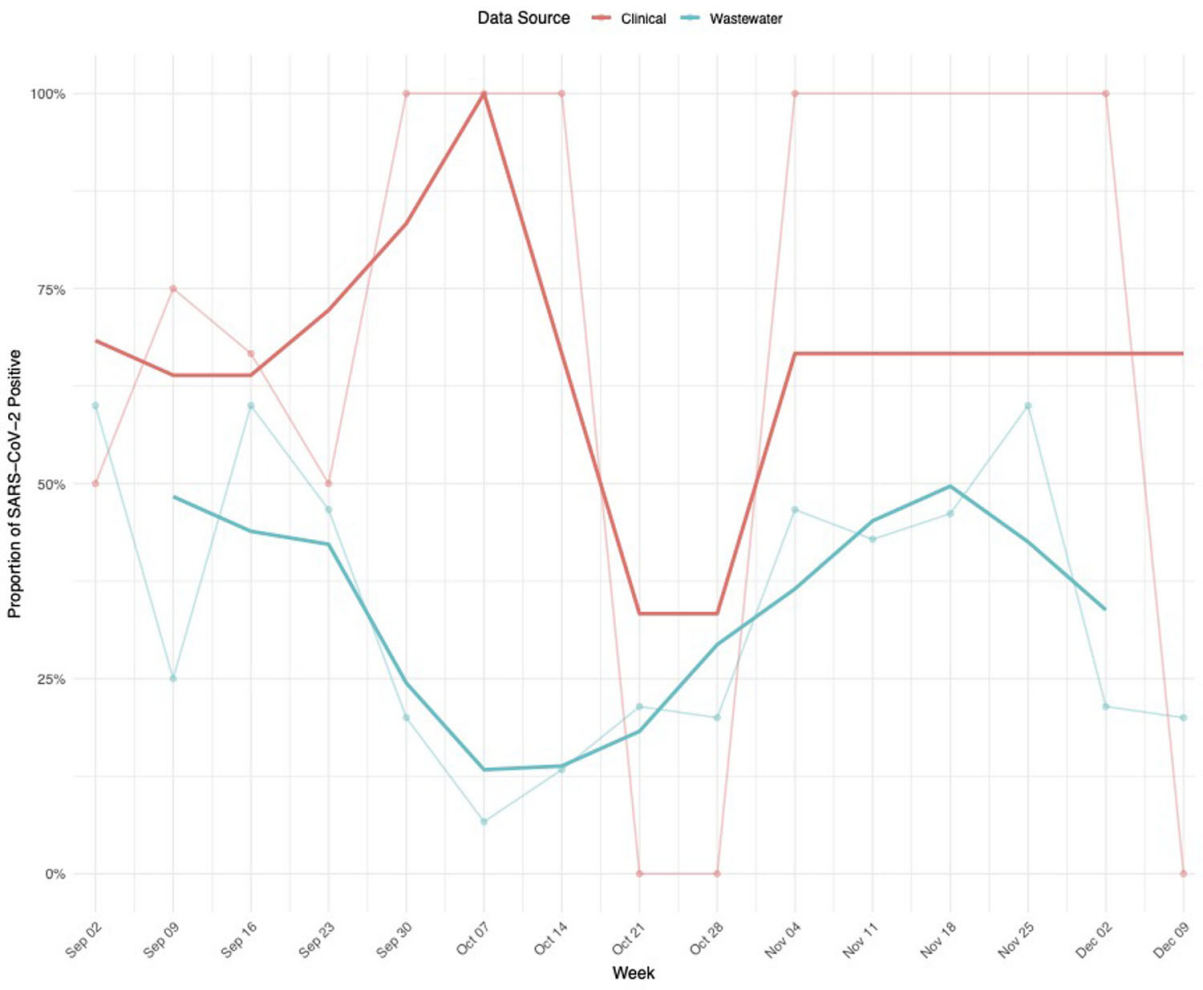

**Figure:**
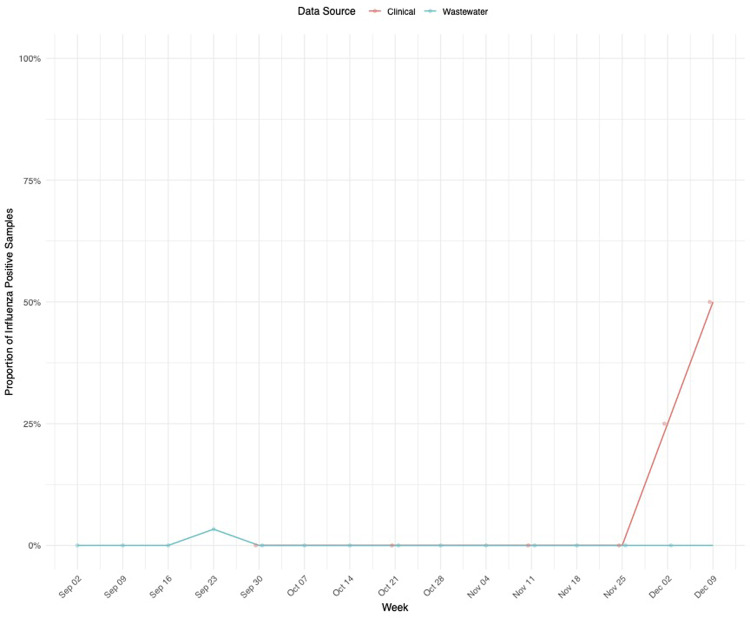

**Figure:**